# Supplementary material for: CellSAM: a foundation model for cell segmentation
Source: Nat Methods. 2025 Dec 8;22(12):2585–93. doi: 10.1038/s41592-025-02879-w (PMC12695629; doi:10.1038/s41592-025-02879-w)
Supplement: Supplementary file 1 — Supplementary Figs. 1 and 2 and Table 1. [file 41592_2025_2879_MOESM1_ESM.pdf]

nature methods

Supplementary information https://doi.org/10.1038/s41592-025-02879-w

CellSAM: a foundation model for cell segmentation

In the format provided by the authors and unedited

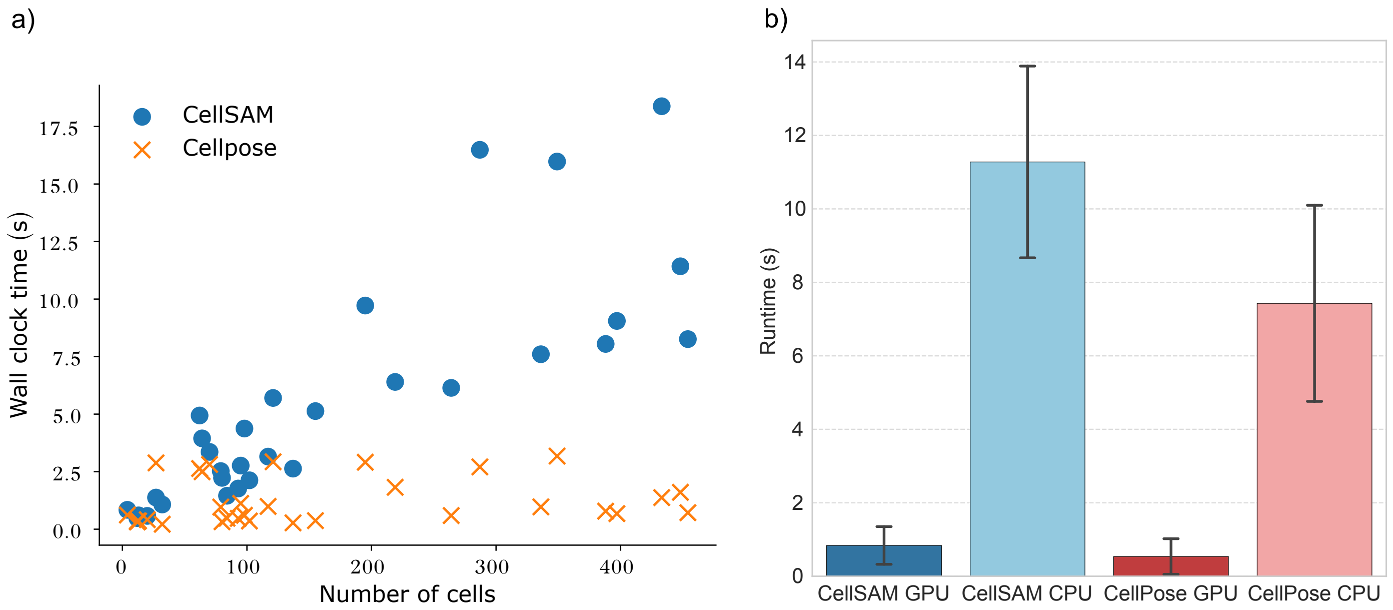

Fig. S1: **Inference times for CellSAM and Cellpose.** a) Wall clock time over the number of cells. Cells were randomly sampled. b) GPU/CPU inference comparison for CellSAM and Cellpose. The Runtime in seconds is per image. N=20 were randomly sampled over multiple datasets. The Bar plot represents the mean and standard deviation.

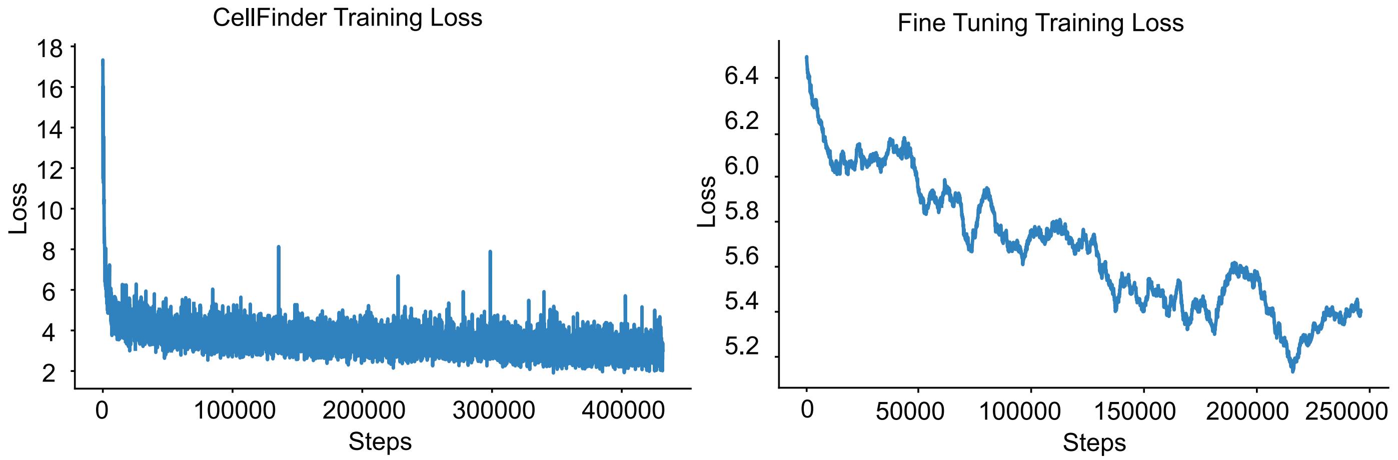

Fig. S2: **Training Curves.** The training curves for training CellFinder (left) and fine-tuning the full CellSAM-generalist model (right) are shown here. We plot the training loss over training steps.

Dataset		Image size [height,width]	Pixel size (µm per pixel)	Test		Train		Val	
				Images	Objects	Images	Objects	Images	Objects
**Tissue**									
	tissuenet_wholecell	[512,512]	0.365∼1.0	330	132568	2576	985134	324	119915
**Cell culture**									
	Cellpose	min:[164,174] max:[576,720]	Various	68	7201	486	58701	54	5978
	BriFiSeg	[512,512]	0.37	22	796	179	6458	23	815
Phase400	3T3	[1608, 1608]	0.55	4	658	32	4106	5	527
	A549		0.55	7	623	60	4950	8	483
	CHO		0.37	4	1002	29	6220	4	883
	HEK293		0.19	2	583	17	4051	3	659
	HeLa		0.65	4	494	32	2789	4	353
	HeLa-S3		0.65	13	3034	103	25421	13	3368
	PC3		0.55	7	696	54	4649	7	571
	RAW264		0.65	13	829	100	6968	13	1214
**H&E**									
	cpm15	min:[392,459] max:[808,1032]	0.38	1	255	12	2048	2	602
	cpm17	min:[500,500] max:[600,600]	0.31	16	1755	32	3766	16	2049
	kumar	[1000,1000]	0.38	0	0	16	9905	0	0
	monusac	min:[90,98] max:[1422,2162]	0.35	21	3864	167	23852	21	3482
	monuseg	[1000,1000]	0.38	7	3822	37	24103	7	2861
	nuinsseg	[512,512]	0.23	1	24	9	325	2	51
	tnbc	[512,512]	0.40	5	391	40	3257	5	408
**Bacteria**									
Omnipose	bact_fluor	min:[335,284] max:[2038,2038]	0.1 or 0.065	23	5388	129	16999	6	691
	bact_phase	min:[66,58] max:[2038,2038]	0.1 or 0.065	145	18400	224	25567	22	1682
DeepBacs	2b_brightfield_dataset	[512,512]	0.14	5	470	2	394	3	425
	2b_fluorescence_dataset	[512,512]	0.14	5	470	2	352	3	425
	2c_e_coli	[1024,1024]	0.045	15	1142	4	335	4	252
	2d_1_SplineDist_dataset	min:[512,512] max:[1024,1024]	0.025	10	649	22	385	10	138
	2d_2_b_subtilis	[1024,1024]	0.065	10	649	70	3843	2	104
	2e_e_coli	[512,512]	0.045	12	688	21	1083	4	269
	s2_stardist	min:[256,256] max:[1024,1024]	0.55	35	2731	120	6656	8	476
**Yeast**									
	YeastNet	[1024,1024]	0.1075	15	468	120	5988	15	894
	YeaZ	min:[512,512] max:[2000,1988]	0.1075	17	1917	303	22526	8	852
**Nuclear**									
	dsb_fixed	min:[256,256] max:[1040,1388]	Various	56	2292	449	20036	57	2558
**NeurIPS challenge**									
	neurips_fixed	min:[200,200] max:[4096,4096]	Various	400	Not publicly available	1099	213683	50	6041

Table 1: Statistics of datasets split by test, train, and val. For computing pixel size, we report values from the dataset source where available. In cases where the dataset does not report pixel size but the imaging camera is known, we report the pixel size of the camera. For other cases, we manually compute an estimation of the pixel size by taking the average major axis length over a subsample of 10 images and dividing by an estimator of the typical length scale for the celltype (e.g., 2 microns for a bacterial dataset). Note that this estimator can have high variance for diverse datasets, e.g., Cellpose, so we do not apply it.
